# Vocal nodules in children: Laryngoscopic morphological classification aids prognostic judgment

**DOI:** 10.3389/fped.2022.941483

**Published:** 2022-09-06

**Authors:** Jia Liu, Wei Cao, Dan-Hua Sun, Lei Wu, Jing Sun, Bin Xu, Yong Fu

**Affiliations:** ^1^Department of ENT and Head and Neck Surgery, National Clinical Research Center for Child Health, The Children’s Hospital of Zhejiang University School of Medicine, Hangzhou, China; ^2^Department of Endoscopy Center, National Clinical Research Center for Child Health, The Children’s Hospital of Zhejiang University School of Medicine, Hangzhou, China

**Keywords:** children, laryngoscopic morphological, therapy choice, vocal nodules, dysphonia

## Abstract

**Objective:**

Vocal fold nodules (VFNs) are benign lesions, occurring at the junction of the anterior and middle thirds of bilateral vocal cords, for which pediatric prognosis and treatment remains controversial. There is a requirement for a correlation indicator to assess the prognosis by pediatric otolaryngologists.

**Materials and methods:**

Patients with VFNs, who were admitted to the department of otolaryngology, were enrolled. The patient’s gender, age, duration of dysphonia, laryngoscopy results and related diseases [allergic rhinitis, sinusitis, laryngopharyngeal reflux (LPR)] were collected and recovery from dysphonia followed up. Correlations between clinical factors and type of laryngoscopic morphology of VFNs were analyzed.

**Results:**

A total of 432 patients with dysphonia were identified, 208 agreed to undergo laryngoscopy and 194 were diagnosed with VFNs as a result. The modal age of patients with dysphonia was between 3 and 7 years with a male: female ratio of 2.1:1 (294:138) and 116 (26.85%; 116/432) had symptoms of allergic rhinitis. The boy to girl ratio among the 194 children with VFNs was about 2.6:1 (140:54) and modal age was consistent with dysphonia. Vocal nodules were the cause of most phonation disorders (93.27%; 194/208) and those with long duration of dysphonia presented more mature (*p* = 0.026) and discrete types (*p* = 0.022). Boys were more likely to have mature (*p* = 0.050) and discrete nodules (*p* = 0.008). Dysphonia recovery time was closely related to age (*p* = 0.000), duration of dysphonia (*p* = 0.000) and morphology of vocal nodules under laryngoscopy (*p* = 0.000).

**Conclusion:**

Vocal nodules are the most frequent cause of phonation disorders in children. The morphological classification by endoscopy may help with clinical diagnosis and therapy choice. In the case of the older child with longer duration of dysphonia and the appearance of mature, large, discrete nodules by laryngoscopy, active treatment is recommended, such as drug therapy, voice therapy or surgical treatment.

## Introduction

Vocal fold nodules (VFNs) are benign lesions occurring at the junction of the anterior and middle third of bilateral vocal cords. VFNs usually present with symmetrical nodular uplift and may result from repeated excessive mechanical vibration of the vocal cord mucosa, hence the phenomenon is also known as vocal nodules or shouting nodules (1). Previous studies have estimated the peak age of onset of vocal nodules as 5–10 years old (2) and an incidence as high as 16.9–30% (3, 4). Pre-disposing factors are considered to be long-term overuse or unscientific methods of vocalization (5). In addition, chronic laryngitis can lead to VFNs (3), a condition caused by allergic reaction (6), laryngopharyngeal reflux (LPR) (7), sinusitis, posterior nostril drip or chronic cough, for example. The current study compares the incidence of VFNs in children of different ages and analyzes the proportion with VFNs in dysphonia. In addition, the effects of allergic rhinitis, sinusitis and LPR disease on dysphonia were assessed by reference to the appropriate epidemiological data.

Controversies surround the prognosis and treatment of VFNs in children. Whereas some physicians believe that children’s vocal nodules will self-heal during adolescence and recommend reduced vocalization and periodic review, the occurrence of dysphonia can have a negative impact on pediatric health. Dysphonia hinders children’s enthusiasm for social interaction and affects self-esteem and self-image formation (8). Thus, there is an argument to be made for aggressive treatment, especially in cases with long recovery times. Correlations among gender, age, duration of dysphonia, related diseases, vocal nodule morphology and prognosis of dysphonia were analyzed for a pediatric cohort. The discovery of clinical indicators with utility for predicting dysphonia recovery time is instrumental in developing personalized treatment plans.

## Materials and methods

### Patients

The patients with dysphonia, who were admitted to the department of otolaryngology of The Children’s Hospital, Zhejiang University School of Medicine (China) between July 1 and August 31 2020, were enrolled. A total of 432 patients with dysphonia were identified, 208 agreed to undergo laryngoscopy and 194 were diagnosed with VFNs as a result. This study was approved by the Medical Ethics Committee of Children’s Hospital affiliated to Zhejiang University Medical College. Data relating to gender, age, duration of dysphonia, laryngoscopy, presence of allergic rhinitis, sinusitis, LPR and other related diseases were collected. Patients were followed up after 1 year to assess recovery from dysphonia.

Laryngoscopy was performed with the informed consent of parents and children. Static images were taken from fiber-optic images of vocal nodules and graded independently by the three pediatric otolaryngologists using a previously validated vocal nodule grading scale (9, 10). Two methods were used for grading (Figures 3–5). (1) A grading scale, based on vocal nodule size and contour (9), devised by Rahul et al., has been clinically validated. Grade 1: small nodules (protrusion<0.5 mm);Grade 2: moderate nodules (protrusion>0.5–1.0 mm);Grade 3: large nodules (protrusion>1.0 mm). Nodule contour may be discrete (nodule base ≤ twice nodule width) or sessile (nodule base ≥ twice nodule width); (2) based on level of nodule maturity (10); immature: local hyperemia, edema and fusiform lesions; mature: fibrotic, white and thin pedicles lesions.

### Clinical characters of dysphonia and vocal fold nodules

Incidences of dysphonia and VFNs were measured. Diagnosis of allergic rhinitis or sinusitis was performed according to the diagnostic criteria of the Guidelines (11, 12). Diagnosis of LPR was performed according to the diagnostic criteria of the Guideline (13).

### Follow up

All patients with dysphonia were recommended to reduce vocalization and shouting and were followed up for 1 year *via* monthly telephone appointments. Final follow-up was completed on December 1 2021.

Briefly, voice recovery was measured over the phone by parents or/and patient including subjective voice assessment. Recovery from dysphonia was graded by parents or/and patient using criteria consisting of five degrees: (1) Complete improvement; (2) Occasional attacks; (3) Frequent attacks; (4) No significant improvement and (5) Aggravation. Occasional attack refers to the occurrence of upper respiratory tract infection causing dysphonia which returns to normal pronunciation after post-infection. Complete improvement and occasional attacks are considered to constitute dysphonia recovery. The time interval from outpatient visit to recovery in months was recorded.

### Statistical analysis

SPSS 22.0 software (IBM Corp., Armonk, NY, United States) was used to assess correlations between clinical factors and laryngoscopic inspection of VFNs by One-way ANOVA, χ^2^-test and Fisher’s exact probability method. The Kaplan-Meier test was used to analyze the correlation between clinical factors and recovery from dysphonia. A *p*-value < 0.05 was considered significant.

## Results

### Clinical data

**FIGURE 1:**
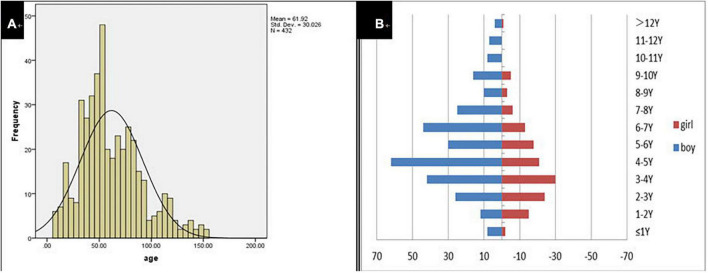
Age distribution of dysphonia **(A)**. Age distribution with dysphonia by gender **(B)**.

A total of 432 children were enrolled in the current study on presentation at the otorhinolaryngology department due to the chief complaint of “dysphonia.” The participants represented 2.37% (432/18,254) of all children who visited the otolaryngology outpatient department during the same period. Participant age ranged from 8 to 153 months with a mean of 61.92 ± 30.03 months. Modal age was between 3 and 7 years. The boy to girl ratio was about 2.1–1 (294–138; Figure 1). The duration of dysphonia ranged from 0.03 to 96 months with a mean of 13.05 ± 15.87 months. Of the total, 116 children (26.85%; 116/432) had allergic rhinitis, 15 (3.47%; 15/432) chronic sinusitis and 17 (3.94%; 17/432) LPR.

### Laryngoscopy results

**FIGURE 2:**
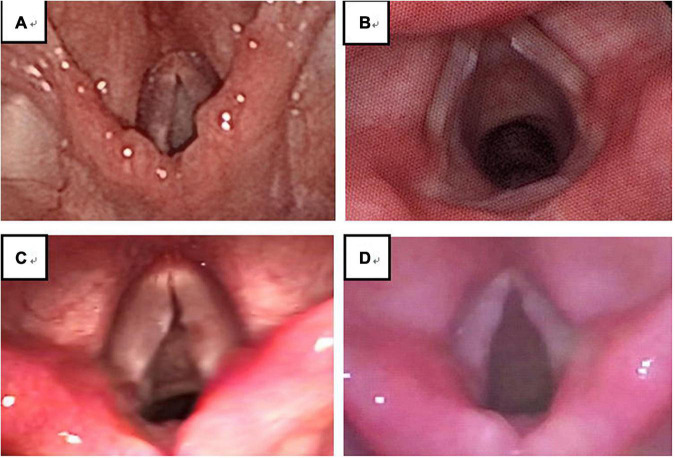
Out of 208 cases, 4 (1.92%) showed no obvious neoplasm **(A)**; among 194 cases of VFNs, 187 cases (96.39%; 187/194) presented bilateral symmetry **(B)** and 7 cases (3.61%; 7/194) presented asymmetry **(C,D)**.

Of the 432 patients treated, 208 agreed to undergo laryngoscopy and 194 (93.27%; 194/208) were diagnosed with VFNs as a result. Of the 194 children with VFNs, the boy to girl ratio was about 2.6:1 (140:54) and the age range was 9–153 months with a mean of 63.86 months (± 25.69). Of the VFNs diagnosed, 187 (96.39%; 187/194) were bilateral symmetry cases and 7 cases (3.61%; 7/194) presented asymmetry (Figure 2).

**FIGURE 3:**
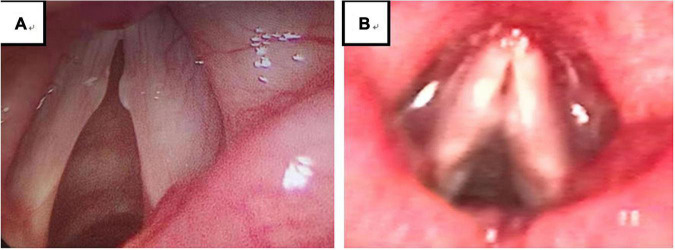
Among 194 VFNs, 90 were immature (46.39%; 90/194) **(A)** and 104 were mature nodules (53.61%; 104/194) **(B)**. Immature nodules: Local hyperemia, edema and fusiform lesions. Mature nodules: Fibrotic, white lesions.

**FIGURE 4:**
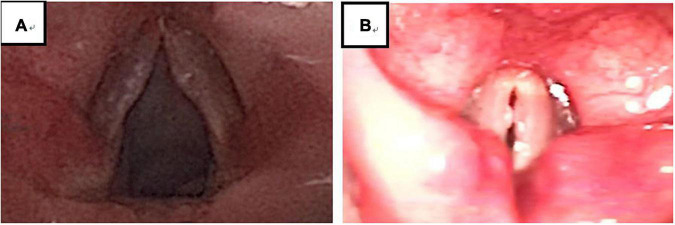
There were 194 VFNs: 99 Sessile (51.03%; 99/194) **(A)** and 95 Discrete (48.97%; 95/194) **(B)**. Sessile: nodule base ≥ twice nodule width; Discrete: nodule base ≤ twice nodule width.

**FIGURE 5:**
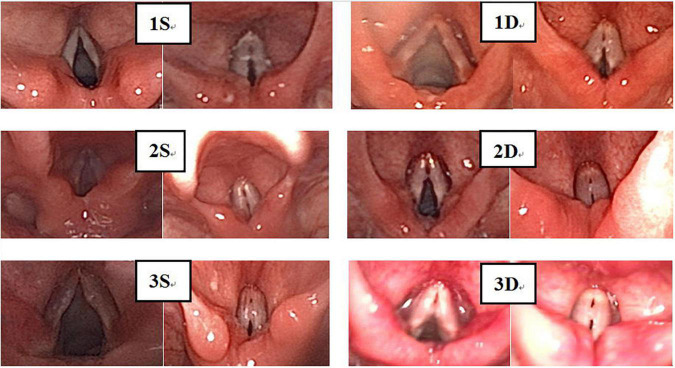
There were 194 cases of VFNs, 76 cases were grade 1 (39.18%; 76/194); 69 were Grade 2 (35.57%; 69/194) and 49 were Grade 3 (25.26%; 49/194). Grade 1: Small nodules (nodule protrudes < 0.5 mm); grade 2: Moderate nodules (nodule protrudes > 0.5–1.0 mm); grade 3: Greater tuberosity (nodule protrudes > 1.0 mm).

Another 14 cases included the discovery from laryngoscopy of laryngitis in 4; papilloma in 2; vocal cord closure gap in 1; vocal cord cyst in 1; vocal cord polyp in 1 and vocal cord paralysis (following neck trauma) in 1. The other 4 cases showed no obvious abnormality by laryngoscopy.

Experienced otorhinolaryngologists were asked to classify vocal nodules based on static fiber-optic images of the larynx. The results showed that 90 cases (46.39%; 90/194) were immature nodules and 104 cases (53.61%; 104/194) were mature (Figure 3); 95 cases (48.97%; 95/194) were discrete and 99 cases (51.03%; 99/194) were sessile (Figure 4); 76 cases (39.18%; 76/194) were Grade 1 small nodules; 69 cases were Grade 2 moderate nodules (35.57%; 69/194) and 49 cases were Grade 3 large nodules (25.26%; 49/194; Figure 5).

### Correlation between laryngoscopic vocal fold nodules morphology and clinical factors

Among the 194 children with VFNs, 42 cases (21.65%; 42/194) were accompanied by allergic rhinitis, 7 (3.61%; 7/194) by chronic sinusitis and 8 (4.12%; 8/194) by LPR. One-way ANOVA analysis indicated that VFNs with long duration of dysphonia tended to present mature types (*p* = 0.026) and boys were more likely to have mature nodules (*p* = 0.050; Table 1). The occurrence of discrete nodules correlated positively with duration of dysphonia (*p* = 0.022) and boys were more likely to present with discrete nodules (*p* = 0.008; Table 2). There was no significant correlation between VFN size and clinical factors or between VFN morphology and allergic rhinitis, sinusitis or LPR.

**TABLE 1:** Correlation between immature/mature VFNs and clinical factors.

Characteristics	Immature nodules	Mature nodules	*F*	*P-value*
Age (month)	90	104	0.847	0.358
Duration of dysphonia (month)	90	104	5.060	0.026
**Sex**				
Male	58	82	7.959	0.050
Female	32	22		
**Companion diseases**				
**Allergic rhinitis**				
Yes	17	25	0.749	0.388
No	73	79		
**Chronic sinusitis**				
Yes	2	5	0.922	0.338
No	88	99		
**Laryngopharyngeal reflux**				
Yes	3	5	0.263	0.609
No	87	99		

Data were statistical analysis by one-way ANOVA, *p-value* < 0.05 was considered significant.

**TABLE 2:** Correlation between VFNs of different sizes or contours and clinical factors.

Characteristics	Vocal nodule size grade			*F*	*P-value*	Vocal nodule contour		*F*	*P-value*
						
	1	2	3			Sessile	Discrete		
Age (month)	76	69	49	0.315	0.730	99	95	0.005	0.941
Duration of dysphonia (month)	76	69	49	1.281	0.280	99	95	5.366	0.022
**Sex**									
Male	54	50	36	0.045	0.956	63	77	7.285	0.008
Female	22	19	13			36	18		
**Companion diseases**									
**Allergic rhinitis**									
Yes	15	17	10	0.282	0.754	19	23	0.783	0.377
No	61	52	39			80	72		
**Chronic sinusitis**									
Yes	2	2	3	0.594	0.553	4	3	0.098	0.754
No	74	67	46			95	92		
**Laryngopharyngeal reflux**									
Yes	2	3	3	0.461	0.631	6	2	1.876	0.172
No	74	66	46			93	93		

Data were statistical analysis by one-way ANOVA, *p-value* < 0.05 was considered significant.

### Recovery time from dysphonia and clinical factors

Of the 432 patients included in the study, 27 were lost to follow-up. Recovery time from dysphonia ranged from 0.07 to 12 months with a mean of 6.66 ± 4.74 months.

Dysphonia recovery time was closely related to age (*p* = 0.000) and duration of dysphonia (*p* = 0.000). A younger age with shorter duration of dysphonia produced a shorter recovery time. Recovery time was not significantly correlated with sex, allergic rhinitis, sinusitis or LPR (Table 3).

**TABLE 3:** Correlation between dysphonia recovery time and clinical factors.

Characteristics	No. (%)	Means of recovery time	χ^2^	*P-value*
**Age (months)**				
≤12	9 (2.08%)	1.814 ± 0.730	34.507	0.000
>12∼≤36	79 (18.29%)	4.956 ± 0.533		
>36∼≤72	201 (46.53%)	7.582 ± 0.333		
>72	143 (33.10%)	7.329 ± 0.392		
**Sex**				
Male	294 (68.06%)	6.515 ± 0.400	0.933	0.334
Female	138 (31.94%)	7.118 ± 0.284		
**Duration of dysphonia (month)**				
≤1	86 (19.91%)	2.937 ± 0.430	115.549	0.000
>1∼≤6	147 (34.03%)	6.485 ± 0.382		
>6∼≤12	97 (22.45%)	7.930 ± 0.467		
≥12	102 (23.61%)	9.784 ± 0.339		
**Companion diseases**				
*Allergic rhinitis*				
Yes	116 (26.85%)	7.567 ± 0.434	2.734	0.098
No	316 (75.15%)	6.688 ± 0.274		
**Chronic sinusitis**				
Yes	15 (3.47%)	6.500 ± 1.167	0.183	0.669
No	417 (96.53%)	6.941 ± 0.236		
**Laryngopharyngeal reflux**				
Yes	19 (4.40%)	7.053 ± 1.112	0.168	0.682
No	413 (95.60%)	6.920 ± 0.237		

Data were presented by Mean ± SD and Kaplan-Meier test. *P-value* < 0.05 was considered significant.

### Recovery time from dysphonia and endoscopic morphology of vocal fold nodules

Of the 194 patients with VFNs included in the study, 12 were lost to follow-up. Recovery time from dysphonia ranged from 0.23 to 12 months with a mean of 7.86 ± 4.31 months.

Dysphonia recovery time was closely related to VFN morphology. Immature (*p* = 0.000), smaller (*p* = 0.000) and discrete nodules (*p* = 0.000) were all associated with a shorter recovery time. Prognostic recovery time was not associated with the occurrence of allergic rhinitis, sinusitis or LPR (Table 4).

**TABLE 4:** Correlation between dysphonia recovery time and VFNs laryngoscopic morphology.

Characteristics	No. (%)	Means of recovery time	χ^2^	*P-value*
**Age (months)**				
≤12	2 (1.03%)	1.250 ± 0.750	14.676	0.002
>12∼≤36	22 (11.34%)	8.023 ± 0.898		
>36∼≤72	109 (56.19%)	8.452 ± 0.410		
>72	61 (31.44%)	8.027 ± 0.549		
**Sex**				
Male	140 (72.16%)	8.374 ± 0.367	0.367	0.545
Female	54 (27.84%)	7.736 ± 0.556		
**Duration of dysphonia (month)**				
≤1	7 (3.61%)	6.571 ± 1.664	8.577	0.035
>1∼≤6	70 (36.08%)	7.388 ± 0.544		
>6∼≤12	46 (23.71%)	7.673 ± 0.667		
≥12	71 (36.60%)	9.495 ± 0.307		
**Companion diseases**				
*Allergic rhinitis*				
Yes	42 (21.65%)	8.404 ± 0.651	0.120	0.729
No	152 (78.35%)	8.144 ± 0.350		
***Chronic sinusitis***				
Yes	7 (3.61%)	6.786 ± 1.684	0.746	0.388
No	187 (96.39%)	8.254 ± 0.312		
***Laryngopharyngeal reflux***				
Yes	8 (4.12%)	7.216 ± 1.788	0.001	0.978
No	186 (95.88%)	8.242 ± 0.311		
**Vocal nodule size grade**				
1	76 (39.18%)	6.032 ± 0.502	32.912	0.000
2	69 (35.57%)	9.642 ± 0.409		
3	49 (25.26%)	9.499 ± 0.568		
**Vocal nodule contour**				
Sessile	100 (51.55%)	5.697 ± 0.401	75.198	0.000
Discrete	94 (48.45%)	10.776 ± 0.289		
**The level of maturity of the nodule**				
Immature	90 (46.39%)	5.328 ± 0.405	97.737	0.000
Mature	104 (53.61%)	10.648 ± 0.282		

Data were presented by Mean ± SD and Kaplan-Meier test. *P-value* < 0.05 was considered significant.

## Discussion

Dysphonia (including hoarseness or rough voice) is a symptom commonly presenting at the otorhinolaryngology department which affects children’s quality of life. As the current study demonstrates, the number of children with dysphonia in the outpatient clinic is large, accounting for 2.37% of all children visiting otorhinolaryngology during the same period. The most common age of presenting children is between 3 and 7 years with a boy to girl ratio of 2.1:1. These observations are consistent with most previous research results (2, 14). Most of the children diagnosed by laryngoscopy had bilateral symmetrical VFNs and age and gender ratios consistent with dysphonia. It has been suggested that VFNs are the most common laryngeal pathological changes in children with dysphonia (9), accounting for 40–62.6% of all causes (2, 15, 16). The current study found an even higher proportion, accounting for about 93.27% (194/208) of total dysphonia. Discrepancies in these findings may be related to parents’ independent choice of laryngoscopy. Children with mild symptoms may be less likely to undergo laryngoscopy, reducing the scope for data collection.

Allergic reaction, sinusitis and LPR have all been identified as being related to the incidence of VFNs (3). Indeed, Ercan et al. (6) proposed that allergic diseases, especially asthma, are significantly correlated with the formation of VFNs. The current study found a large number of children with allergic rhinitis (26.85% with dysphonia and 21.65% with VFNs). However, dysphonia recovery time and VFN morphological classification were not significantly correlated with allergic rhinitis. Ercan et al. show that effective asthma control is essential for VFN treatment and it suggested that the effect of lower respiratory allergic disease on VFNs may be greater than that of allergic rhinitis. No significant correlations were found between sinusitis or LPR and VFNs. Sinusitis and upper respiratory tract infections are closely related in children, although there are fewer sinusitis attacks during the hot summer period of data acquisition for the present study. LPR is difficult to diagnose in children, due to the difficulty of performing relevant examinations, resulting in a high false negative rate of the disease.

Treatment of VFNs in children remains controversial. Ongkasuwan et al. (17) observed that the coordination of children’s treatment was poor and, in combination with a tendency for self-healing during adolescence, observation and follow-up were suggested. Furthermore, Choi and Cotton (18) and Mori (19) believed that non-surgical treatments could delay the course of the disease for months to years, resulting in a severe psychological and economic burden on the children and their parents. Surgical treatment can lead to a short-term improvement in symptoms. Levine et al. (20) and Hooper (21)advocated appropriate administration of antibiotics and steroid hormones plus ultrasonic atomization inhalation to ameliorate the condition. More recently, many experts have recommended voice training (22, 23). Nonetheless, long-term and chronic dysphonia has a negative effect on children’s lives (24), generating an urgent need for relevant reference indicators to guide the formulation of individual treatment plans.

Many previous studies have investigated morphology and voice analysis indices in the context of VFNs (10, 25). However, few studies have attempted to correlate VFN morphology, clinical factors and dysphonia recovery time. The current study used static fiber-optic images of children’s larynxes to grade nodules according to maturity, size and contour. The results indicated that prolonged mechanical vibration and friction promotes nodule maturation, producing a pale, tough and discrete morphology but not affecting nodule size. No correlation could be found between VFN morphology and allergic rhinitis, sinusitis or LPR, suggesting that the local inflammatory state has no obvious influence on VFN morphology. Positive correlations were found between age, duration of dysphonia and dysphonia recovery time. Mature large discrete nodules tend to require a longer recovery time. In summary, if the child is older, has a longer duration of dysphonia and laryngoscopy shows mature, large, discrete nodules, active treatment is recommended, such as drug therapy, voice therapy or surgical treatment. In the absence of active treatment, observation and follow-up are required.

We acknowledge some limitations to the current study. The evaluation of children with dysphonia is largely subjective, produced by children and parents, and no objective index of voice assessment at initial evaluation and follow-up exists. Laryngoscopy was not performed during follow-up reviews since parents and children were unwilling to undergo the procedure. Thus, no data was available to compare dysphonia recovery with VFN morphology recovery. The current data were collected intensively to draw prognostic conclusions. Further study of different treatment options is still required.

## Data availability statement

The original contributions presented in this study are included in the article/supplementary material, further inquiries can be directed to the corresponding author.

## Author contributions

JL: drafting the manuscript, conception, and design of study. WC, JS, and BX: acquisition of data. D-HS and LW: analysis and/or interpretation of data. YF: revising the manuscript critically for important intellectual content. All authors contributed to the article and approved the submitted version.
